# Human milk microbiota: what did we learn in the last 20 years?

**DOI:** 10.20517/mrr.2022.05

**Published:** 2022-05-25

**Authors:** Marta Selma-Royo, Joaquim Calvo-Lerma, Christine Bäuerl, Maria Esteban-Torres, Raul Cabrera-Rubio, Maria Carmen Collado

**Affiliations:** Department of Biotechnology, Institute of Agrochemistry and Food Technology, Spanish National Research Council (IATA-CSIC), Valencia 46980, Spain.

**Keywords:** Human milk, microbiota, infant health

## Abstract

Human milk (HM) is the gold standard for infant nutrition during the first months of life. Beyond its nutritional components, its complex bioactive composition includes microorganisms, their metabolites, and oligosaccharides, which also contribute to gut colonization and immune system maturation. There is growing evidence of the beneficial effects of bacteria present in HM. However, current research presents limited data on the presence and functions of other organisms. The potential biological impacts on maternal and infant health outcomes, the factors contributing to milk microbes’ variations, and the potential functions in the infant’s gut remain unclear. This review provides a global overview of milk microbiota, what the actual knowledge is, and what the gaps and challenges are for the next years.

## WHAT IS KNOWN ABOUT HM MICROBIOTA?

Human milk (HM) is the first food ingested by a newborn. It provides optimal nutrition during the first months of life, being the gold standard for infant nutrition. In terms of its composition, HM contains a unique and optimal combination of nutrients and bioactive components, including immunoglobulins and cytokines, bioactive lipids, oligosaccharides, microRNAs, hormones, and microorganisms, among others. The concentrations of these substances vary among individuals, within the lactational stage, within a day, and between feeds. The concentrations are also determined by diet, maternal genotype, gestational age, maternal health status, and the environment around the mother^[1-6]^. Thus, HM has a complex and unique composition that fulfills infants’ needs and supports their growth and development.

The complexity of HM and the factors that contribute to shaping its composition are essential aspects that need to be considered to expand the knowledge about HM. To date, most of the HM studies have focused on nutritional factors and on identifying the bacteria that may play a role in infant gut colonization^[7-9]^. However, there is significant interest in microbiota-related research, aiming to identify specific microorganisms, microbial molecules, and metabolites that contribute to various aspects of the host’s physiology and health. Technical, methodological, and biological issues need to be further addressed, and many fundamental questions about HM microbial ecosystems remain to be answered. Further research is required to determine whether specific bacteria, along with lifestyle, contribute to the maintenance of microbial equilibrium, as well as the potential functionality of milk microbes for infant health and host-microbe interactions.

In this review, we aim to summarise the actual knowledge on HM microbiota, as well as provide a global perspective on the knowledge gaps and the limitations of milk microbiota studies.

## HM MICROBIOTA ANALYSIS: PITFALLS AND LIMITATIONS IN TECHNICAL ASPECTS

There are specific limitations in studying HM microbiota including the higher intra- and inter-variability in microbial communities, driven by maternal, neonatal, and environmental factors. We also discuss the lack of information on viability and activity of the milk microbes, the problems associated with the management of low microbial biomass samples, the limited use of negative and positive controls, the lack of procedure standardization (differences in sampling, processing, DNA extraction, amplicon region, sequencing platform, *etc*.) and also, the heterogeneous study designs and lack of functional studies among others.

The viability of the milk microbes has been widely studied as the potential benefits have been associated with potential adhesion to the neonatal gut, immune stimulation, production of short-chain fatty acids, and other activities that require viable bacteria. Traditional microbiological techniques have provided a diverse range of cultivable bacteria in HM, including *Staphylococcus*, *Streptococcus*, and related Gram-positive genera, such as lactic acid bacteria and bifidobacteria, *Rothia*, and others. Overall, more than 200 bacterial species belonging to approximately 50 different genera have been isolated from HM^[10]^, and the use of culturomic approaches is increasing these numbers rapidly^[11]^. Culture-independent techniques, specifically next-generation sequencing techniques, have greatly helped to increase the knowledge about the microbial ecosystem of HM, although the viability of those microbes cannot be tested. These analyses allow an extraordinary degree of detail in the analysis of microbial diversity; however, this approach has its limitations, such as PCR amplification biases^[12]^, which may underestimate the number of Gram-negative bacteria. This amplification bias is well known in some groups of bacteria that are not easily amplified by universal primers, such as *Bifidobacterium* (containing high G + C content), which can make a difference in the gut microbiota of healthy infants^[13]^. *Bifidobacterium* and other bacteria are also difficult to lyse due to cell-wall composition; the use of enzymatic treatments and bead-beater increase the DNA efficiency, but this is not always performed. We cannot forget that there are several aspects that limit the interpretation of 16S rRNA-derived results. The most important is the fact that its copy numbers per genome vary from 1 up to 15 or more copies^[14,15]^. Another important factor is the effect of 16S rRNA region choice on bacterial community metabarcoding results^[16]^. Although this all adds imprecision to the analyses, it is necessary to work in an adequate way, as well as to explain that they are not quantitative or exact and work with the same hypervariable zone. On the other hand, although there are programs to solve these errors^[17]^, there are manuscripts that do not advise the normalization of the number of copies of the 16S rRNA gene since it does not provide more reliable conclusions in metataxonomic studies^[18]^. What is interesting is knowing the best way to process the data to minimize these biases^[13]^. Finally, other limitations are inherent in the different or specific hypervariable regions of 16S rRNA targeted by sequencing^[19]^.

High-throughput sequencing provides a powerful window into the structural and functional profiling of microbial communities, but it is unable to characterize only the viable (or active cells) portion of microbial communities at scale. To profit from the potential advantages of next-generation sequencing and include the viability issue, other techniques based on RNA (meta-transcriptomics, active cells), the use of viability kits (e.g., LIVE/DEAD) coupled with flow cytometry, and the use of propidium monoazide as a fast way to obtain DNA from intact cells (viable cells) have been developed^[20,21]^.

Another limitation of the study of HM is the low biomass, which poses some challenges to researchers due to the risk of DNA contamination. Careful precautions are essential to avoid contamination and to identify microbial DNA signals from the environment or extraction and sequencing kits^[22,23]^. We found a few studies with recommendations to reduce this risk^[24-26]^, such as the improvement in DNA extraction protocols. It is necessary to include negative controls, as well as to identify and eliminate contaminating sequences during the bioinformatic analysis^[27-29]^, which should be considered in studies of the milk microbiome.

One of the major limitations that we can face when writing a review on HM microbiota involves the high variations in the studies dealing with this topic and their heterogeneous designs. These may partially be explained by sample collection protocols (aseptic or non-aseptic procedure), sample storage (4 ºC, -20 ºC, -80 ºC), and processing (whey milk or whole milk), as well as the DNA extraction procedures (e.g., different commercial kits, use of columns, enzymatic or mechanical lysis, *etc*.). The greatest difference among these studies is the collection of samples (some aseptically and others using different procedures). To avoid contamination of the sample from skin or milk extraction devices, conservation protocols are convenient^[30]^. All these sources of variability need to be reported, including whether specific procedures have been used to avoid skin and environmental microbiota, or this was also considered in the analysis as part of the microbial load that the breastfed infant received. Recently, a unified guideline for reporting microbiota studies has been proposed, the STORMS checklist^[31]^. These general recommendations will facilitate the results reproducibility, a better interpretation of HM microbiota analysis, and will reduce the heterogeneous designs in future studies.

Another limitation depends on the sequencing platform used, length of reads, and/or the specific bioinformatic analysis, as well as the multiple software programs, pipelines, and databases used in the analysis. These results are often reported at different taxonomic levels (i.e., phylum, family, genus, and species), making comparison among studies difficult. Additionally, many of the studies do not report the use of antibiotics during pregnancy, during delivery, or while breastfeeding. Microbiota analysis techniques also vary considerably among the HM studies. Furthermore, multiple maternal and environmental factors affect the milk microbiome, which, together with the small number of samples analyzed in most of the available studies, makes it difficult to draw biologically significant and universally valid conclusions. Therefore, it is necessary to define the inclusion/exclusion criteria and the collection of adequate metadata (e.g., the maternal diet) to ensure that the biological data obtained answer the specific question being investigated.

The majority of the studies about HM microbiota are based on 16S rRNA profiles (i.e., amplicon-based sequencing, targeted qPCR). Thus, knowledge at the functional level in this environment that could be obtained with metagenomic approaches (e.g., shotgun total DNA sequencing) is still lacking. Considering what occurred in other environments, such as the gut, microbial functions could be better biomarkers for health status compared to taxonomic composition. In HM, there are hardly any metagenomic studies that enable the full characterization of the functional capacity; however, this challenge is real and enormous. Shotgun sequencing (metagenomics) in HM would imply great challenges, such as how to manage contaminating DNA from the host, low biomass samples, and computational analysis (taxonomic, functional, assembly, contigs, *etc*.). However, if the inconveniences can be overcome, the metagenomic analysis would reveal new aspects in the HM research, such as the knowledge about new functions of interest from non-culturable bacteria in complex communities^[32]^ and improvement in the resolution of the taxonomic assignment. Indeed, metagenomics could be useful in the tracking of the vertical transmission of specific strains from the mother to the neonate^[33,34]^, although the full potential of this methodology in the HM analysis has not yet been explored. With this methodology, not only is the bacterial community observed, but also other types of populations, such as eukaryotic, prokaryotic, and viral, increasing the knowledge of HM globally. These populations have already been studied previously with more specific approaches (e.g., amplifying a specific gene; metataxonomic), focusing on yeasts, viruses, and fungi^[35-39]^. It is necessary to add that all these DNA-based studies provide information on the composition of the community and its metabolic potential, but they do not demonstrate the activities that they carry out *in situ *because it only predicts the functional capacity from the taxonomic composition. To partially overcome such limitations, there are other alternatives such as metatranscriptomic^[40]^, metaproteomic^[41]^, or metabolomic^[42]^ methods. Each of these methodologies would include several different challenges. However, they must be faced in order to acquire more knowledge about the complexity of HM and its relation to different variables, such as time, health, delivery, weight, stress^[1]^, allergy^[43]^, and so on, as well as how to affect the microbiota with other HM components, such as oligosaccharides and lipids^[44]^.

## HM MICROBIAL COMPONENTS: WHAT ARE THE RELATIONS AMONG THEM?

Some decades ago, HM was considered a sterile fluid. The first microbiological studies on HM focused on the detection of harmful microbes and their role as sources of breast or infant infections. However, over the last two decades, several studies have revealed the existence of a low biomass site-specific microbiota in the pre-colostrum, colostrum, and mature milk of healthy women^[45-47]^. The evidence of the presence of potentially beneficial microorganisms in HM, such as *Lactobacillus* and *Bifidobacterium*, started to emerge during the first decade of this century^[46,48]^. Since then, it has drawn microbiologists’ attention to the microbiological characterization of HM. HM microbiome research has increased since those first studies, and the use of next-generation sequencing has expanded our knowledge over the past 20 years. We summarise the knowledge in this section.

### Bacteria

A systematic review summarised the results presented in publications focusing on HM microbiota until 2019; the results showed that 590 genera and 1300 species were identified in HM^[49]^, with the median bacterial load between 10^5^ and 10^6 ^cells/mL^[39]^. HM microbiota was generally composed of Firmicutes and Proteobacteria phylum, while Actinobacteria is present in lower relative abundance. The most abundant genera found in the HM were *Staphylococcus* and *Streptococcus*, whose presence seemed to be present in almost all samples^[49]^, followed by *Pseudomonas*, *Lactobacillus*, *Bifidobacterium*, and *Corynebacterium* genera. However, there is still a high variability in the results reported by Zimmerman and Curtis, probably due to the variability in the experimental design. Other systematic analyses revealed that the most detected species were facultative anaerobic or strictly aerobic such as *Staphylococcus aureus*, *Staphylococcus epidermidis*, or *Streptococcus agalactiae*^[50]^. The same authors found in their literature review that highly oxygen-sensitive species have also been reported in HM analysis, but only by culture-independent analysis. However, the confidence about the presence of anaerobic bacteria in the HM is still uncertain since several issues are faced in the analysis of these taxa including the intrinsic limitations of culture-independent techniques regarding the viability of the detected species and the usual collection methods. Further studies with new experimental procedures are needed to decipher the potential presence of these taxa in the HM as well as their potential function in the infant’s gut.

### Yeast and fungi

A fungal fraction, usually neglected, is gaining interest in determining the mycobiota fraction in HM^[38,51-54]^. The presence of viable fungi in HM has been confirmed by culture-dependent and independent methods^[39]^, with *Malassezia*, *Candida*, and *Saccharomyces*^[51]^ identified as the most prevalent taxa. Indeed, HM mycobiota showed a specific, different composition, as well as higher fungal diversity and richness compared to the surfaces of the hospital neonatal units^[54]^. Although the fungal component has been known for a few years, the bacteria-fungi interaction is usually neglected in microbiome studies, with just two of them exploring this relation, to our knowledge^[55,56]^. It has been identified milk bacterial taxonomic clusters with differences in their levels of HM fungi. For example, enrichment in the Proteobacteria phylum was observed in those samples with no presence of fungi^[55]^, suggesting a negative relationship between this phylum and fungi prevalence. A correlation between bacterial and fungal richness and a co-exclusion association between the *Candida* genus and some bacterial genera were also found. Another study likewise showed a complex network of intradomain and interdomain interactions between bacterial and fungal taxa, which would be influenced by geographical location, delivery mode, and some maternal features^[56]^. Among others, the geographical location would influence the presence, quantification, and composition of HM mycobiota^[55,56]^. Further studies are needed to clarify the interactions between HM bacterial and fungal populations and their roles in infant development.

### Virus and phages

Human virome, defined as comprising all viruses found in the human body, has been even less characterized. Although just a few studies are available^[57-59]^, HM virome shows a specific composition that differs from other body sites^[58]^. Bacteriophages, specifically the Myoviridae, Siphoviridae, and Podoviridae families, are the most abundant viruses in HM^[58,60]^. As these families have mainly lytic natures, these bacteriophages could affect and regulate the composition of bacterial microbiota^[61]^, establishing a relation between the phages and the bacterial population in HM and, consequently, in the infant’s gut. Maqsood *et al*. confirmed the dominance of the mentioned bacteriophages but found Herpesviridae as the most relatively abundant virus in HM^[57]^. Other studies did not confirm the association between HM bacteriophages and bacterial communities^[58]^. It has been reported that HM is a transmission source of viruses, which could modulate the infant colonization process^[58]^. Specific vertical transmission of bifidobacterial phages has also been reported^[62,63]^. However, the HM virome’s potential role in infant development is totally unexplored to date. From previous studies unrelated to HM, the association between the virome and health status is well-known. A relative resilience of the HM virome composition in HIV-infected women has been described^[57]^; however, gut virome was altered in malnourished infants^[64]^ and also in adults with irritable bowel syndrome^[65]^. In this regard, eukaryotic viruses could have a direct impact on infants’ innate and adaptive immune system maturation^[66]^. These, along with the bacteriophages’ capacity to modulate the microbiome composition, highlight the virome as an essential determinant of infant development that needs to be assessed to a greater extent in microbiome studies. Since only a few studies have focused on HM, further research is needed to decipher its virome diversity and the factors that could influence it, as well as the impacts of these changes on maternal and infant health.

### Archaea

Despite the few available studies, the presence of archaea has also been identified in HM^[67,68]^. Even though its contribution to milk microbiota is low, seven archaeal genera and species have been identified in raw bovine milk samples, with *Methanobrevibacter* and *Methanosarcina *being the most abundant genera^[67]^. Archaea have also been identified in HM microbiota by metagenomics sequencing and have been associated with the control groups in the context of mastitis studies^[38]^. However, only one study has explored the viability of archaeal components in HM, to our knowledge^[68]^. In this cited study, the authors were able to isolate viable methanogenic archaea, mainly *M. smithii*, in almost half of the analyzed samples from both colostrum and later milk. The authors also described the detection of *M. smithii* by qPCR in approximately one-third of the mothers included in the analysis (*n *= 127). Indeed, the frequency of this detection was higher in overweight compared with normal-weight mothers, suggesting a potential link between the archaea in HM and maternal metabolic condition.

### Interactions between HM microorganisms and HM bioactive compounds

As mentioned, it has been hypothesized that milk components are possibly interacting in the HM ecosystem and thus, influencing one another. In fact, some perinatal factors that have been reported to affect HM microbiota such as lactation stage, geographical location, delivery mode, maternal body mass index (BMI), and diet^[69,70]^ also influence mycobiota composition^[55,56]^, HM virome^[60]^, macronutrients content^[71,72]^, human milk oligosaccharide (HMO) profile^[73,74]^, and immune components^[75-77]^. However, studies addressing these links are scarce. Specific associations between lipid content and HMO profile with microbiota composition and diversity have been reported^[44]^. Other associations among HM microbiota (both compositional and bacterial load), macronutrients, and human somatic cell counts have been reported^[39]^.

Although the available data are still limited, more studies using a multiapproach design that target more than one HM component would be essential for a more compressive analysis that provide more practical conclusions. Thus, a better understanding of the microbial interactions with the other HM components and how maternal factors could affect this network would increase the knowledge about HM dynamics, with the translational potential in the field of infant nutrition.

## HM MICROBIOTA: WHAT OCCURS DURING GASTROINTESTINAL DIGESTION?

The field of food science has traditionally focused on characterizing the proximal composition of foods and the effects of industrial manufacturing or processing. However, this approach has failed to consider an unavoidable step that eventually transforms foods and defines the extent to which food components are available for biological functions, the digestion process. Research in recent years has established that the physical structure of the food matrices and the gastrointestinal environment of the individuals entail an interaction that conditions the bioaccessibility of nutrients contained in foods^[78-80]^. Therefore, currently, the concept of a food’s nutritional value cannot be established without considering the transformations imparted by the digestion process.

In this sense, HM, as a food matrix is one of the most complex; it is a source of not only nutrients but also immunoglobulins and microorganisms, among other components. Thus, establishing the biological value of HM should address not only the bioaccessibility of its nutrients, but also the persistence of immunoglobulins and the survival of microbes after the different stages of the digestion process in which these components are relevant. Hence, we bring into the concept that changes in the amount and type of HM microbes could result from the process of digestion, and this could be studied using similar research approaches to those applied to follow bioaccessibility of HM nutrients during digestion. Posing this research question from the perspective of “what occurs during digestion” could shed light on relevant aspects of the newborn’s growth, including the establishment of the immune system, or the first colonization and establishment of the colonic microbiota. However, the current scientific literature contains few reports on the transformation of HM after the digestion process. The scarce evidence could be related to the difficult access to gastrointestinal contents, in which the digestion phenomenon takes place. Although taking fecal samples as substrates to perform the pertinent digestibility analyses is a valid approach, the *in vitro* simulation of the digestion process is also widely implemented and accepted^[81]^. In fact, there are specific protocols in the laboratory to reproduce the digestion process in different physiological contexts. In particular, digestion in lactating infants has been parametrized in different protocols, having in common its shorter duration, less acidic gastric pH, and lower concentration of enzymes compared with the protocols established for adults^[82]^. In the following paragraphs, we compile and summarise the available *in vivo* and *in vitro* studies.

Addressing the bioaccessibility of nutrients and bioactive compounds in milk is probably the most studied topic in this sense. Among the nutrients in HM, oligosaccharides and lipids have attracted the most attention because of their implications for growth. However, most of the available studies are designed with the goal of improving the quality of infant formulas rather than providing scientific evidence of HM digestion. For example, using the infant TIM-1 *in vitro* digestion model, Fondaco *et al*. studied the different lipolysis patterns in infant formulas compared with HM, showing the higher bioaccessibility of lipids in HM^[83]^. A number of authors reached the same conclusions^[84,85]^, reporting the bioaccessibility of lipids in HM at around 85%, and others focused on following the bioaccessibility of specific fatty acids, such as docosahexaenoic acid^[86]^. A relevant remark in this sense is that apart from lingual and pancreatic lipase, lipolysis in lactating infants includes the bile salts-stimulated lipase. This lipase is secreted at the mammary gland and remains inactive during gastric digestion, and resumes its activity at the intestinal stage when in the presence of bile salts^[87]^. Besides, HM fat globules present different structure and composition than that in infant formulas^[83]^, so both are relevant aspects to consider when following lipid digestion with *in vitro* digestion models. In terms of protein, another *in vitro* digestion study explained that proteolysis did not differ among the goat-based infant formula, the cow-based infant formula, and HM, but the kinetics of protein digestion of the goat-based formula was more comparable to that of HM^[88]^.

Regarding carbohydrates, HM oligosaccharides are the most interesting species because of their potential role as prebiotics. In this sense, their inalterability through oral, gastric, and small intestine digestion has been confirmed for two decades, verifying their availability as substrates for the microbiota at the colonic stage^[89]^. In this case, the *in vivo* confirmation of this finding is available, as their presence in fecal samples has been identified^[90]^. However, no study has specifically focused on the presence of undigested macronutrients in fecal samples of breastfed infants.

Focusing on the immunogenetic role of HM, immunoglobulins are some of the main responsible agents. However, these molecules are also susceptible to being degraded throughout gastrointestinal digestion. Immunoglobulins A (IgA) and G (IgG) are known to have a local effect at the intestinal epithelium level, as established for intestine-secreted IgA^[91]^. However, when the immunoglobulin origin is HM, some IgG and IgM could be partially degraded in the gastric or intestinal digestion step. While immunoglobulins could play a relevant role in the oral cavity, their potential effect on the small intestine has been shown to decrease in the contexts of the respiratory syncytial virus^[92]^ and the SARS-CoV-2^[93] ^infections. This information could explain the HM components’ contribution to the infant’s immune system, especially regarding the role of agents other than immunoglobulins, if these are certainly degraded through digestion. Current evidence, however, indicates that at least maternal IgA, which is mostly bound to secretory components, is protected from digestion and binds bacteria in the infants’ intestine. Indeed, according to Brandtzaeg *et al*., maternal IgA is the only immunoglobulin present in the infants’ intestine^[94]^. Currently, no other studies related to immunoglobulins degradation during digestion have been identified in the literature.

Finally, HM microorganisms could suffer a reduction of viability, as reported in experiments on the degradation of microorganisms after digestion in other food sources related to fermented dairy products^[95]^. While an extended research line in the follow-up on probiotic strains’ survival is well-established^[96-98]^, very few studies address the viability of the microbiome in HM as a whole. This is precisely the aspect that could have the most impact in the study on the primary gut colonization, as there is a current gap in this regard. In fact, an immense number of studies characterizing the microbiota profile in HM (at different lactation times, ethnicities, geographical locations, maternal diets, *etc*.) are increasingly available^[2,69,99,100]^, but none of them have included research on the viability of the microorganisms after digestion.

Therefore, we have identified the need to conduct studies on the survival of HM microbiota after the digestion process in order to establish or quantify the extent to which HM microbiota remains available for setting up infants’ colonic microbiota and thus, apply a better approach to determining the real role of HM microbiota in developing infants’ microbiota. However, we acknowledge some limitations in assessing the viability of bacterial cells after simulated digestion, especially in the design of experimental protocols, including the determination of the optimal milk sample dilution and the potential issues of the toxicity of the digesta in intestinal cells, which make it difficult to analyze the epithelial adherence.

## HM MICROBIOTA-HOST INTERACTIONS

### What is the main origin or source of milk microbes?

For years, researchers have tried to explain the potential origin of HM microbiota, and the debate is still open. Different routes and hypotheses have been suggested: (1) the environment, including the mother’s skin; (2) breast tissue, which harbors bacteria as other human epithelia^[101]^; (3) maternal gut and oral microbiota through an endogenous route^[102-105]^; or (4) retrograde translocation^[106]^. Finally, other microbial sources include the skin as well as the oral cavity of newborns that have been exposed to the mother's vaginal and intestinal microbiota during delivery^[34,107]^. This hypothesis has been less considered since pre-colostrum produced by women during their first pregnancies contains bacterial strains that can be isolated from the mouths of their neonates after being breastfed^[47]^. In fact, these observations also suggested a potential role of HM in oral colonization. However, the available evidence revealed that this reverse flow of bacteria could occur during breastfeeding and their implication in HM seeding could not be discarded^[106]^.

The most studied route of transmission is the transfer of the bacteria present in the human intestine and/or oral cavity to the mammary gland through an endogenous route and several mechanisms supporting this route have been proposed. One of them would involve the lymphoid system associated with the mucosa; however, the components implicated in this translocation and how the processes occur have not been deciphered^[103,108]^ and many questions remain with no answer^[106]^. Another mechanism behind the endogenous route would be related to the endocytosis of bacterial cells, which could be supported by some physiological responses observed in late pregnancy. These include the altered tight junction regulation in the gut during this period and the effect of birth-associated stress on intestinal permeability^[109,110]^. Bacterial interactions with the host’s immune system appear to differ among various sites in the body, and understanding how the maternal immune system interacts with milk bacteria can help elucidate the mechanisms that allow a potential bacterial translocation from the gut^[111]^ and/or the oral cavity. Although several routes have been described, the available literature does not enable to clarify the contribution of each of those mechanisms in the HM microbiota seeding, and even a combination of all of them in a multi-origin of the HM microbiota could not be discarded.

Therefore, more questions than answers remain in the field, and specific research addressing these questions would expand our knowledge of the HM microbiota origin and, more relevantly, how we could affect the process of its bacterial seeding.

### Microbial vertical transmission

The composition of the infant gut microbiota is dependent on many factors, being those together with other bioactive factors present in the HM (e.g., HMOs and immune components), important for the modulation of the infant gut microbiota composition and functionality in early life^[112-116]^. Gut microbiota plays an important role in immune system development, protecting against pathogens and facilitating the digestion and absorption of nutrients, with breastfeeding as the most influential factor affecting infant gut microbiota development^[113]^. Thus, milk microbiota may be responsible for many of the short-/long-term health-promoting effects associated with exclusive breastfeeding in early life^[115,117,118]^, as it would correlate with reduced incidence of chronic inflammatory and metabolic conditions in infancy and adulthood^[119]^.

In recent years, the HM microbiota composition has been extensively studied in an attempt to unravel its role in infant and maternal health. However, the mechanisms that facilitate the establishment and stability of the gut microbiota in early life, as well as its association with favorable health outcomes, remain poorly understood^[120]^. In fact, as was mentioned, most of the available studies in the field have been performed using a 16S rRNA sequencing approach, which does not enable the strain-level tracking needed to unravel the specific role of HM bacteria in infant colonization. All these raise new pivotal research questions about the function of milk bacteria in the establishment and functionality of infant gut microbiota^[106]^, in which studies based on a metagenomic approach would be key. 

Besides this, the study of host-milk microbes is crucial^[121]^. The main limitation in deciphering the interactions between milk microbiome and human intestinal cells is the scarcity of bacterial isolates of milk origin^[122]^. Therefore, more advances in culture-dependent approaches and bacterial cell viability analysis will be of key importance in identifying and isolating the viable part of the milk microbiota for functional analysis. Another important limitation is that the few studies about shared taxa vertically transferred from mothers to babies through breastfeeding have been performed using molecular approaches^[33,46,115,123-129,130]^. The interest in this topic has increased considerably in recent years; a recent large cohort study (CHILD) highlights the low co-occurrence of bacterial species in mothers’ milk and their infants’ stools^[115]^. These studies suggest that the milk could provide pioneering species to the infant’s gut, but the mentioned studies mainly focus on the sharing of individual taxa without exploring the global impact of milk bacteria on the overall infant gut microbiota^[115]^. Additionally, the studies employed the 16S rRNA sequencing, which is not reliable in characterizing microbial taxa at the species/strain level and even less in identifying their function. These limitations are more evidenced by the limited number of bacterial species isolated from maternal milk and infant feces, belonging to *Bifidobacterium, Staphylococcus, Lactobacillus*, and* Escherichia*/*Shigella*.

Thus, the available data highlights that the composition of HM microbiota differs from infant gut microbiota. Although the dominated bacterial taxa present in HM don’t colonize the infant’s gut efficiently, they would be able to optimize and modulate the proliferation of pioneer bacteria which uniquely participate in host interactions^[116]^.

In this context, more and larger longitudinal cohorts analyzing the vertical transfer of microbiota via breastfeeding, coupled with the application of high throughput sequencing technologies and metabolomics with a wide range of microbial culturing conditions, will significantly improve the knowledge about HM microbiota colonizing the infant gut^[131]^.

### What is the relevance for infant gut health?

The complex interplay among milk microbiota, immune constituents, and infant gut colonization is of great importance. However, the question of whether the bacteria present in milk are established in the gut, interact with host cells, and influence the offspring’s physiology remains poorly understood. The gut microbiota can affect the host in multiple ways, such as the production of antimicrobial compounds and metabolites (i.e., vitamins, aromatic acids, and short-chain fatty acids)^[132-134]^, or with the presence of extracellular components (e.g., exopolysaccharides, pili, *etc*.), which add more complexity in carrying out mechanistic approaches.

To date, only a few HM-related microbial components involving host-microbiota interactions are currently known. It is well-established that breastfeeding provides prebiotic HMOs to support the developing infant gut microbiota^[135,136]^. In this regard, most of the research has focused on *Bifidobacterium*, since some strains efficiently utilize the HMOs present in milk^[137]^; thus,it is assumed that *Bifidobacterium* spp. are evolutionarily selected to be transferred to the infant and have co-evolved with their infant host^[62,127]^.

The protections against pathogens by the production of acetate or aromatic lactic acids are some mechanisms exerted by milk bacterial components. In fact, several of the bacteria shared between HM and the infant’s gut are involved in lactate production (*Bifidobacterium*, *Streptococcus*, and *Staphylococcus*). *Bifidobacterium* spp. also produce the short-chain fatty acid acetate through saccharolytic fermentation of oligosaccharides^[138]^. Collectively, acetate and lactate promote the low pH in the gut, which protects against infections and facilitates the transport of acetate into the gut epithelium to be used by the host^[138]^. Furthermore, specific milk isolates (*Lactobacillus* ssp. and *Staphylococcus epidermidis*) have been shown to produce bacteriocins^[10,139]^; for this, milk commensals were postulated as a strategy against the growth of gut pathogens^[10]^.

A few studies have shown that some milk bacteria may participate in the maturation of the infant immune system through the modulation of both natural and acquired immune responses in mice models and humans. For example, *Lactobacillus salivarius* CECT 5713 and *Lactobacillus fermentum *CECT 5716 have a broad array of effects on the immune system, enhancing the production of pro- and anti-inflammatory cytokines and chemokines, activated NK cells, CD4+ and CD8+ cells, and regulatory cells^[10,140,141]^. In line with this, HM *Lactobacillus* strains produce butyrate, which is an important regulator in the infant’s gut as well as an energy source for colonocytes^[142,143]^. More studies using bacteria of milk origin will increase the knowledge of the cross-talk between other milk microbiota strains, the gut epithelium^[144]^, and immune components. For example, the analysis of the interaction between IgA and milk bacteria, as studied in *Bacteroides fragilis* (and other gut commensals), showed a mediated stable gut colonization through the exclusion of pathogen competitors^[145]^. Another limitation of these studies, which is common in gut microbiota analysis, is that the effects are generally analyzed in stool samples instead of *in situ* in the gut epithelium.

A better understanding of the dynamics and function of milk microbiota requires a comprehensive multi-pronged approach that assesses the viability and activity of milk bacteria, evaluates the interactions between milk microbiota and maternal and infant immune systems, and experimentally establishes the functional significance of milk microbiota. Emerging *in vivo *animal and human studies offer novel opportunities to address the gaps in this field, contributing to identifying the mechanisms governing the milk microbiota assembly and its impacts on maternal and infant health.

### How are host-microbes interactions analyzed?

Alternatives to human observational or epidemiologic studies and animal models in gut microbiota research are certainly *in vitro*/*ex vivo* models using human cells in order to replace, reduce, and refine animals. Cancer cell lines have been widely used due to their robust, often indefinite growth and cost-effectiveness; however, their origin limits the research questions that can be addressed. One of the most popular cell lines is Caco-2, which exhibits characteristics of small intestinal epithelial cells despite being derived from a colon carcinoma^[146]^. In an attempt to use cell lines that have a greater resemblance to the immature infant intestine, foetal epithelial intestinal cell lines such as FHs74Int and H4 emerged^[147,148]^. Particularly, H4 cells served as a model for necrotizing enterocolitis to study probiotics or HM-derived components after an inflammatory insult^[149-151]^. Human intestinal organoids that were derived from either adult stem cells or pluripotent stem cells emerged in the last decade as useful model systems. This technique requires microinjection of microbiota into the organoids’ lumen in order to maintain the 3D structure. Furthermore, the availability of nutrients in the lumen is limited, which only allows a short exposure time to living bacteria^[152,153]^. To overcome these drawbacks, confluent 2D intestinal cell monolayers can be generated from single-cell suspensions of enzymatically dissociated organoids^[154]^. To date, intestinal organoids have been used more for studying HM components, such as short-chain fatty acids and indole-3-lactic acid^[150,155]^, than for studying HM-derived bacteria. Noel *et al*. applied colostrum whey milk to paediatric enteroid monolayers and found enhanced tight junction function, increased production of the antimicrobial peptide a-defensin 5 by goblet and Paneth cells, and increased levels of polymeric immunoglobulin receptor and maternal IgA translocation^[156]^. However, to study the effects of HM or derived components or bacteria, more sophisticated *in vitro* model systems should be used, such as gut chips^[157,158]^ or gut organ culture systems^[159]^ combining several cell types present in the intestine (i.e., epithelial, endothelial, and immune cells and bacteria) and considering oxygen concentrations gradient and peristaltic movement, thus resembling more physiological conditions.

Due to the lack of studies analyzing the interactions between milk microbiota (and other bioactive compounds) and the mammary gland, it would be interesting to use 3D cell culture models of mammary glands, such as organoids or mammary glands on a chip, that provide complex interactions and mimic physiological conditions of in vivo experiments more faithfully^[160-162]^.

In order to advance in mechanistic insights into milk microbiota host-interactions, researchers should combine animal *in vitro* and *in vivo* ( mice, piglets, *etc*.) methods that will deliver information to establish accurate *in vitro*/*in vivo* extrapolation to humans^[163]^. In line with this, translational research is needed and essential.

## WHAT IS THE SCIENTIFIC EVIDENCE OF THE HM’S IMPACT ON MATERNAL-INFANT HEALTH OUTCOMES?

HM confers both nutritional and immunological benefits to neonates, which are significant due to the macronutrients and bioactive components discussed above. Most importantly, breastfeeding reduces morbidity and mortality in infants, such as a two-fold lower risk of death observed in exclusively breastfed infants compared with non-breastfed infants^[164]^. Several studies have documented lower incidences of diarrhea and respiratory infections, as well as protection against ear, throat, and sinus infections^[165,166]^. Among HM’s components, the microbiota has received special attention among researchers who aim to discover its potential role in the mentioned protective effects of breastfeeding. Studies comparing breastfed versus formula-fed infants suggest a vertical mother-to-infant microbial transmission through breastfeeding^[112,167,168]^. The contribution to infant microbial seeding could be one of the pathways through which HM microbiota impacts infant health^[169]^.

Other indirect mechanisms have also been suggested, including the facilitation of gut epithelium maturation through mucus production and the decrease in intestinal permeability^[155]^ or the immunomodulatory properties of some HM strains^[170]^. Despite the observational studies reporting the protective effects of breastfeeding, it is still difficult to find a direct link between HM microbiota and some of these benefits. Other milk components, such as antimicrobial peptides, immune active compounds, growth factors, or microRNA, could also be partially responsible for these associations^[171,172]^. Therefore, further studies are needed to ascertain the impacts of HM microbiota on maternal and infant health and to understand their interactions with the other bioactive components of milk. Thus, in this section, the generally beneficial effects of breastfeeding on both maternal and infant health are presented.

In this context, attention must be paid to preterm infants; necrotizing enterocolitis is the most devastating disease among the most premature infants. HM diet has been shown to decrease the relative risk of necrotizing enterocolitis and is considered one of the strategies for its prevention^[173]^. Preterm infants have commonly shown a dysbiotic microbiota pattern loaded with pathogenic bacteria that could be implicated in the necrotizing enterocolitis development^[174,175]^. Besides the potential impacts of HM’s nutritional and anti-inflammatory functions that could decrease the necrotizing enterocolitis risk, the positive implications of some beneficial bacteria and related metabolites present in HM have also been suggested^[150,176,177]^. Thus, some clinical studies have reported reductions in necrotizing enterocolitis incidence and severity^[178]^ after probiotics administration. However, the evidence is still considered inconclusive due to the lack of trials with large sample sizes^[179]^. Prolonged and exclusive breastfeeding has also been suggested to improve infants’ cognitive development^[180,181]^. It has been associated with fewer autistic traits^[182]^ and increased verbal than non-verbal skills^[183]^. However, the results are still contradictory, and potential confounding factors could not be discarded^[184,185]^.

Associations between infant gut microbiota composition and diversity and lower scores on the visual reception scale and in expressive language at the age of 2 years^[186]^; communication, personal, and social skills at the age 3 years^[187]^; and general neurodevelopment^[188]^ have been described. Despite the potential influence of HM microbiota on infant colonization, specific links between these observations and HM are still unknown. Although several mechanisms have been proposed^[189]^, further studies are needed to understand the pathways behind the effect of HMO on infant neurodevelopment.

There is still conflicting evidence on the protective role of breastfeeding in relation to the development of allergic disease and asthma later in life. However, different meta-analyses have concluded that a longer duration of breastfeeding is associated with reduced risks of wheezing and asthma, allergic rhinitis in children up to 5 years old, and eczema in children up to 2 years old^[190,191]^. HM microbiota plays a key role in shaping early immune development and response; therefore, it has been suggested as a tool for allergy prevention^[192]^. Most of the proposed mechanisms to explain the latter effect are based on the impact of breastfeeding on infant gut microbiota, but the specific effect of the HM microbial population is not well-known yet^[193]^. In this sense, children developing allergic manifestations at 7 years of age consumed HM with a reduced microbial richness in the first month^[194]^. As HM and infant microbiota are modifiable factors, the administration of probiotics and prebiotics is under study in pregnant and lactating mothers, as well as in neonates, with promising results for risk reduction in allergic complications later in life. Nevertheless, the evidence is still scarce, and larger, well-standardized studies are needed^[195-197]^.

There is also evidence that breastfeeding protects against obesity in childhood^[198-200]^ and is reported to be influenced by the child’s sex, maternal education, maternal BMI, excessive gestational weight gain, maternal smoking, and maternal alcohol consumption^[198,199,201]^. Similar to the potential link between milk microbiota and allergy, the potential of HM microbiota to drive gut colonization is suggested as the mechanism behind the mentioned association between breastfeeding and lower obesity incidence. Indeed, several studies have shown the shifts in infant microbial community as related to an overweight condition, and some molecular mechanisms have been proposed, such as enhanced energy intake, fat storage and low-grade systemic inflammation, which could be all microbially induced^[202]^. The latter hypothesis is supported by studies showing that antibiotic use in infants during breastfeeding increase the obesity risk^[168,203]^, highlighting the potential microbial component as one pathway through which it exerts its beneficial effects on infant health.

### FUTURE PERSPECTIVES AND CHALLENGES

It is assumed that HM plays an important role in seeding the infant gut microbiome pioneers. However, many of the bacteria detected in milk samples have been reported as absent in the infant’s gut; in contrast, the most abundant bacteria in the infant’s gut, *Bifidobacterium*, have been found only in < 40% of HM samples, suggesting that HM may act as an additional source of colonization^[106,204]^. In this regard, more metagenomic and bacterial isolations could help determine the role of milk microbiota in infant gut microbial colonization, since some bacterial groups could be underestimated with the current analysis. Indeed, these studies could also help unravel the potential connections between and among different components of HM microbiota, such as the fungi-bacteria interaction, and how they could affect the milk composition.

To date, research on milk-microbiota interactions has been mainly conducted with mono-colonized models using vertically transferred bacteria or those of non-milk origin. It would be interesting to study the mechanisms of action of a consortium of milk-related bacteria. Therefore, the development of novel *in vitro* (e.g., co-culture of microorganism consortia with living epithelial cells using conventional culture techniques, organs on chip models, and organoids), *ex vivo*, and *in vivo* models of mammary glands and intestines is essential to shedding new light on the field of milk microbiota. Moreover, most of the studies have not considered the effect of the host in order to draw a complete map of host-microbe interactions. Thus, the proposed approaches will reveal the bacteria present in infant microbiota whose origin is maternal milk, their potential biological functions, and bacteria-produced bioactive compounds of relevance to infant health. These approaches will additionally decipher the molecular mechanisms underlying host-microbe interactions in infants, not only in the gut but also in other niches, such as the oral and nasopharyngeal microbiota, which could have implications for infants’ systemic health and development.

### Challenges for the next years

- To conduct studies that include interactions among microorganisms from HM beyond the bacterial component. Explore their relations with other milk bioactive constituents (nutrients, hormones, cytokines, *etc*.) and how the variation could affect the HM populations.

- How do milk bacteria alter milk composition before reaching the infant’s gut? Study the HM as a whole ecosystem with interactions among its components. Conduct multiapproach and analytical studies.

- To improve and standardize HM collection protocols, use more aseptic HM collection methods.

- To detect and reduce sequencing artifacts.

- To analyze the translocation of gut bacteria to HM via the enteromammary pathway, which would provide opportunities for improving infant health through maternal interventions.

- To decipher the contribution of infant oral bacteria to milk microbiota.

- To develop more advances in shotgun sequencing, metabolomics, and metatranscriptomics, which are needed to establish exactly if shared bacteria are functionally significant to the infant.

- To study the relevance of milk microbiota to the development of the microbiota in other body locations. 

- To apply 3D cell models and novel animal models (piglets) to the analysis of milk microbiota-host interactions.

- To conduct mechanistic studies to analyze the association of milk/gut microbiota with infant health.

- To explore the role played by non-viable bacteria in HM.

- To establish the effect of the HM digestion process on viable microorganisms reaching the colon.

- To promote the translational research and global system integration

## DECLARATIONS

### Copyright

© The Author(s) 2022.
